# First Error-Based Supervised Learning Algorithm for Spiking Neural Networks

**DOI:** 10.3389/fnins.2019.00559

**Published:** 2019-06-06

**Authors:** Xiaoling Luo, Hong Qu, Yun Zhang, Yi Chen

**Affiliations:** School of Computer Science and Engineering, University of Electronic Science and Technology of China, Chengdu, China

**Keywords:** spike neural networks, supervised learning, synaptic plasticity, first error learning, speech recognition

## Abstract

Neural circuits respond to multiple sensory stimuli by firing precisely timed spikes. Inspired by this phenomenon, the spike timing-based spiking neural networks (SNNs) are proposed to process and memorize the spatiotemporal spike patterns. However, the response speed and accuracy of the existing learning algorithms of SNNs are still lacking compared to the human brain. To further improve the performance of learning precisely timed spikes, we propose a new weight updating mechanism which always adjusts the synaptic weights at the first wrong output spike time. The proposed learning algorithm can accurately adjust the synaptic weights that contribute to the membrane potential of desired and non-desired firing time. Experimental results demonstrate that the proposed algorithm shows higher accuracy, better robustness, and less computational resources compared with the remote supervised method (ReSuMe) and the spike pattern association neuron (SPAN), which are classic sequence learning algorithms. In addition, the SNN-based computational model equipped with the proposed learning method achieves better recognition results in speech recognition task compared with other bio-inspired baseline systems.

## 1. Introduction

For years, researchers have been exploring and trying to simulate the brain's powerful and high-speed information processing capabilities and learning mechanisms. While the traditional artificial neural networks (ANNs) have achieved outstanding performance in various application fields, they assume that sensory information is represented and transmitted via the firing rate of the neuron. Nevertheless, the rate-based coding does not seem to transmit all the information associated with the rapid processing sensory tasks, such as vision, smell, and hearing stimulus modalities (Hopfield, 1995; Gautrais and Thorpe, 1998; Cariani, 2004; Mohemmed et al., 2013). A new type of artificial neural network that is dedicated to the study of more biologically plausible neuronal models and neural networks has emerged and has been well used (Wu et al., 2018a,b), which is called spiking neural networks (SNNs). On the other hand, many recent studies have shown that spike-timing neural activities exist in several areas of the brain, such as the visual cortex (Bair and Koch, 1996), the retina (Meister, 1998; Uzzell and Chichilnisky, 2004; Gollisch and Meister, 2008), and the lateral and geniculate nucleus (Reinagel and Reid, 2000). Temporally encoded SNNs that represent information as precisely timed spikes rather than mean firing rates have also been studied extensively (Maass, 1997; Andrew, 2002; Ghosh-Dastidar and Adeli, 2009b; Nguyen et al., 2012; Wang et al., 2012). Though the powerful computing performance of SNNs has been demonstrated (Keller and Hahnloser, 2009), its practical application is still limited by its computational complexity, and the learning algorithms applicable to SNNs are also generally short of high efficiency and stability. Therefore, it is of great significance to develop new effective and robust learning algorithms to take full advantage of the powerful computing performance of SNNs.

In many cases, learning behavior is thought to be performed by utilizing the error signals, i.e., the mismatches between expected and actual spiking behaviors (Thach, 1996; Bastos et al., 2012; Keller et al., 2012; Wu et al., 2019). Supervised learning based on error signals has obtained the most documented evidence in the study of the cerebellum and cerebellar cortex of the central nervous system, although the exact mechanism still remains unclear (Ito, 2000). The aim of supervised learning is to minimize the gap between actual output and expected output, and according to the different ways of reducing the gap, the existing learning algorithms of SNNs can be divided into two categories. One is to utilize rigorous mathematical analysis to derive formulas of loss reduction, and the other is to make weight updating according to the inspiration of biological mechanisms, such as the Widrow-Hoff rule (Widrow and Lehr, 1990) and the spike-timing dependent plasticity (STDP) rule (Masquelier et al., 2009), where the synaptic strength is enhanced when the presynaptic neuron elicits spikes before the postsynaptic neuron and vice versa.

Many methods based on mathematical analysis adopt the idea of gradient descent, but they define the cost function in different ways. SpikeProp (Bohte et al., 2002) minimizes the loss defined by the distance between the true firing time and the single desired firing time using gradient descent rule, and later this algorithm was improved to emit multiple spikes (Ghosh-Dastidar and Adeli, 2009a; Xu et al., 2013a). In addition to these methods, Tempotron (Gütig and Sompolinsky, 2006), an algorithm that has been proved to be effective for binary temporal classification but unable to handle the firing of multiple spikes, and some other algorithms (Zhang et al., 2018, 2019a) define the cost function as the distance between the membrane voltage and the firing threshold. Recently, there is another thought of defining cost function of multi-spike sequences. For example, Multi-Spike Tempotron (MST) (Gütig, 2016) is designed to decrease the difference between a hypothetical threshold and the fixed threshold. MST also employs the gradient descent strategy, and in each iteration the difference between the fixed biological firing threshold and the hypothetical threshold under which neurons emit the expected amount of spikes is calculated. However, it requires multiple recursive calculations to derive the hypothetical threshold, making the learning process indirect and computationally time-consuming. TDP1 and TDP2 (Yu et al., 2018) simplify the calculation of MST to some extent, which improves the learning efficiency, but there is still the problem of seeking the hypothesis threshold through iteration.

The Remote Supervised Method (ReSuMe) (Ponulak and Kasiński, 2010) is a classic algorithm that combines the STDP and anti-STDP learning rules to modulate the synaptic weights. There are also some improved algorithms to further strengthen the learning property of the ReSuMe by integrating it with delay learning (Taherkhani et al., 2015a,b, 2018), and particle swarm optimization (PSO) algorithm (Xie et al., 2014), etc. In addition, the Spike Pattern Association Neuron (SPAN) (Mohemmed et al., 2012), Chronotron E-learning (Florian, 2012), and the Precise-Spike-Driven (PSD) (Yu et al., 2013) algorithm are in a similar vein, whereby they transform spike trains or sequences into analog signals by convolution, then apply the Widrow-Hoff rule to update weights. SPAN uses a variant metric of the van Rossum metric (van Rossum, 2001) to define the distance between the actual and desired spike sequences, while Chronotron E-learning uses the Victor and Purpura metric (Victor and Purpura, 2009). SPAN transforms all the discrete input, actual and desired output spikes to continuous signals, while only input signals are convolved in PSD. Compared with algorithms requiring convolution operation, algorithms based on the perceptron rule, such as the perceptron-based spiking neuron learning rule (PBSNLR) (Xu et al., 2013b) and its improved version (Qu et al., 2015), the normalized perceptron based learning rule (NPBLR) (Xie et al., 2017), are easier to calculate. In general, these algorithms are more biologically plausible and have lower computational complexity than the algorithms based on the gradient descent rule, but they are still not very effective and robust in the task of learning target spatiotemporal spike patterns.

Except for these algorithms, the algorithm Learning Spike Sequences with Finite Precision (FP) (Memmesheimer et al., 2014) uses the existing postsynaptic potential to adjust the synaptic weights at the first unmatched time between the actual and desired output spike trains in each trial. However, the simple and crude way of weight modification makes it use less spike information and also lack good robustness in the face of noise. Then in this paper, we propose a new efficient and robust learning algorithm. The proposed algorithm not only utilizes the first wrong spike time, but also utilizes all previous spike temporal information to calculate the weight update quantities. Simulation results demonstrate that the proposed learning rule has higher learning accuracy, efficiency, and better robustness as compared with ReSuMe and SPAN. In addition, in this paper, we also put forward a dynamic decoding strategy for precise multi-spike learning algorithms. With a combination of the proposed learning algorithm and the decoding strategy, the SNN-based computational model outperforms other bio-inspired baseline systems in a speech recognition task.

The structure of the article is as follows. In 2, after a brief introduction of the neuron model, our method is presented. In 3, we conduct some experiments to explore the performance of the method, and the simulation results are provided. The different properties of the proposed algorithm, ReSuMe and SPAN are analyzed and compared in 4. Finally, we draw the conclusion in 5.

## 2. Neuron Model and Learning Algorithm

In this section, we first introduce the spiking neuron model used in this article, then elaborate on the algorithm we proposed. Finally, the measurement used to evaluate the learning performance is introduced.

### 2.1. Neuron Model

Many spiking neuron models have been proposed over the years, among which conductance-based models can simulate biological neurons' dynamics accurately to a large extent but require considerable computational cost because of the inherent complexity of their expressions. By contrast, the current-based leaky integrate-and-fire (LIF) (Gerstner and Kistler, 2002) model can well simulate the dynamics of biological neurons with lower computation cost, which has made it a widely used model in many papers, including this one.

In the LIF model, learning neuron accumulates its membrane voltage *V*(*t*) by integrating synaptic currents from *N* upstream neurons, yielding

(1) $$\begin{array}{l} V\left(t\right)=\overset{N}{\underset{i=1}{\sum }}w_{i}\underset{t_{i}^{j}<t}{\sum }K\left(t-t_{i}^{j}\right)-\vartheta \underset{t_{s}^{j}<t}{\sum }\exp\left(-\frac{t-t_{s}^{j}}{\tau_{m}}\right), \end{array}$$

where $t_{i}^{j}$ is the firing time of the *j*th spike from the *i*th synapse and $t_{s}^{j}$ is the firing time of the *j*th spike generated by the learning neuron. ϑ is the firing threshold. *w*_*i*_ represents the synaptic strength of the *i*th synapse, and it controls the amplitude of the postsynaptic potential induced by its spike, while the kernel *K*(·) controls the shape, and it is defined as

(2) $$\begin{array}{l} K\left(x\right)=V_{norm}\left[\exp\left(-\frac{x}{\tau_{m}}\right)-\exp\left(-\frac{x}{\tau_{s}}\right)\right], \end{array}$$

where τ_*m*_ and τ_*s*_ are the time constants of the membrane potential and the synaptic current, respectively. *V*_*norm*_ is the normalization constant that stretches the peak value of *K*(·) to unit, and it is calculated by

(3) $$\begin{array}{l} V_{norm}=\frac{\beta^{\beta /\left(\beta -1\right)}}{\beta -1}, \end{array}$$

with β = τ_*m*_/τ_*s*_. If the voltage *V*(*t*) reaches the firing threshold, it triggers a spike immediately, then this new spike causes the membrane voltage of the neuron to encounter a reset operation, which is expressed by the second term in Equation (1).

### 2.2. First Error Learning Algorithm

The aim of our learning algorithm is to modify the neuron's synaptic weights so that it can generate the target spike sequence corresponding to the given input spike pattern. Most existing algorithms train the neuron to fire spikes directly toward the desired times, but here we set a tolerance window with a small width ε (less than the distance between any two desired spike times) at each desired time, and by training the neuron to emit a spike within the corresponding tolerance window in chronological order, the requirement of firing target spike sequence is finally achieved. Accordingly, we present our learning method taking advantage of the idea of running synaptic modification rules only at the first wrong spike time in each trial in Memmesheimer et al. (2014).

**Figure 1:**
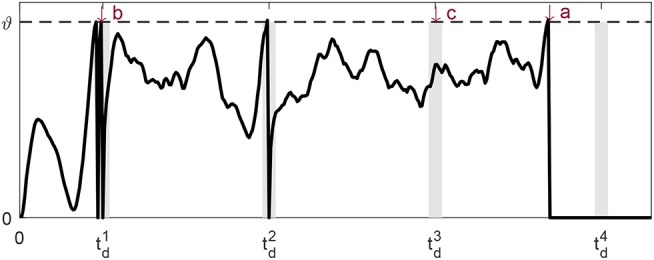
Three error types: undesired spike outside the tolerable window **(a)**, undesired spike inside the tolerable window **(b)** and missed spike within the tolerable window **(c)**. The gray vertical bars near the desired spike times $t_{d}^{j}$ are the respective tolerable windows.

There are different types of wrong spike times, but in general they all fall into one of the three categories and are shown in Figure 1:

- If there is a spike fired outside all tolerable windows, this spike time is a wrong spike time of type a;
- If there are two spikes generated within a same window, the second spike time is a wrong spike time of type b;
- If there is no spike within the desired tolerable window, the desired spike time is a wrong spike time of type c.

Following the idea of running synaptic modification rules only at the first wrong spike time in each trial, the proposed First Error Learning rule (FE-Learn) calculates weight adjustment in a new way that utilizes more temporal information between the input and output spike trains. Based on the different error types, the proposed method employs two weight updating processes. The cost function is defined as

(4) $$\begin{array}{l} E=\pm \left(\vartheta -V\left(t_{err}\right)\right), \end{array}$$

where *t*_*err*_ is the first wrong spike time and the ± sign corresponds to weight increment and decrement, respectively.

#### 2.2.1. Weight Increment at Desired Output Spike Times

In terms of error type c, a spike is supposed to be emitted within the tolerable window of a desired output spike time $t_{d}^{j}$, while it is not, so *t*_*err*_ is equal to $t_{d}^{j}$. Then, we apply the gradient descent method to stretch the membrane potential at time *t*_*err*_ to the threshold ϑ.

In gradient-based learning, the weight modification Δ*w*_*i*_ is proportional to the negative of the derivative of the cost function with respect to *w*_*i*_:

(5) $$\begin{array}{l} \Delta w_{i}=-\lambda_{1}\frac{dE}{dw_{i}}=\lambda_{1}\frac{dV\;\left(t_{err}\right)}{dw_{i}}, \end{array}$$

where λ_1_ > 0 is the learning rate that defines the size of the weight increment. From Equation (1), the membrane potential *V*(*t*_*err*_) not only receives the direct influence of the synaptic weights, but also the indirect influence of them, which is transmitted by the previous output spike times $t_{o}^{j}<t_{err},j=1,2,\cdot \cdot \cdot \;,m$. The derivative term in Equation (5) is hence given by

(6) $$\begin{array}{l} \frac{dV\;\left(t_{err}\right)}{dw_{i}}=\frac{\partial V\;\left(t_{err}\right)}{\partial w_{i}}+\overset{m}{\underset{j=1}{\sum }}\frac{\partial V\;\left(t_{err}\right)}{\partial t_{o}^{j}}\frac{dt_{o}^{j}}{dw_{i}}. \end{array}$$

From Equation (1), the first term of Equation (6) can be expressed as

(7) $$\begin{array}{l} \frac{\partial V\;\left(t_{err}\right)}{\partial w_{i}}=\underset{t_{i}^{j}<t_{err}}{\sum }K\left(t_{err}-t_{i}^{j}\right), \end{array}$$

and the partial derivative in the second term is

(8) $$\begin{array}{l} \frac{\partial V\;\left(t_{err}\right)}{\partial t_{o}^{j}}=-\frac{\vartheta }{\tau_{m}}\exp\left(-\frac{t_{err}-t_{o}^{j}}{\tau_{m}}\right), \end{array}$$

while for the derivative $dt_{o}^{j}/dw_{i}$, applying the chain rule, we can get

(9) $$\begin{array}{l} \frac{dt_{o}^{j}}{dw_{i}}=\frac{\partial t_{o}^{j}}{\partial V(t_{o}^{j})}\frac{dV(t_{o}^{j})}{dw_{i}}\\ \;=\frac{\partial t_{o}^{j}}{\partial V(t_{o}^{j})}\left(\frac{\partial V(t_{o}^{j})}{\partial w_{i}}+\sum_{k=1}^{j-1}\frac{\partial V(t_{o}^{j})}{\partial t_{o}^{k}}\frac{dt_{o}^{k}}{dw_{i}}\right)\\ \;\approx \frac{\partial t_{o}^{j}}{\partial V(t_{o}^{j})}\frac{\partial V(t_{o}^{j})}{\partial w_{i}}, \end{array}$$

in order to save the computation cost, we eliminate the iterative computation term in Equation (9). Following the linear assumption of threshold crossing in Bohte et al. (2002), Ghosh-Dastidar and Adeli (2009a), and Yu et al. (2018), the neuron's membrane potential is thought to increase linearly in the infinitesimal time step before the firing time. Hence, there is

(10) $$\begin{array}{l} \frac{\partial t_{o}^{j}}{\partial V\left(t_{o}^{j}\right)}=-{\left(\frac{\partial V\left(t_{o}^{j}\right)}{\partial t_{o}^{j}}\right)}^{-1}, \end{array}$$

where

(11) $$\begin{array}{l} \frac{\partial V(t_{o}^{j})}{\partial t_{o}^{j}}=\frac{\partial V(t)}{\partial t}{|}_{t=t_{o}^{j-}}\\ \;=\frac{V_{norm}}{\tau_{s}}\sum_{i=1}^{N}w_{i}\sum_{t_{i}^{j}<t_{o}^{j}}\exp\left(-\frac{t_{o}^{j}-t_{i}^{j}}{\tau_{s}}\right)\\ \;-\frac{V_{norm}}{\tau_{m}}\sum_{i=1}^{N}w_{i}\sum_{t_{i}^{j}<t_{o}^{j}}\exp\left(-\frac{t_{o}^{j}-t_{i}^{j}}{\tau_{m}}\right)\\ \;+\frac{\vartheta }{\tau_{m}}\sum_{k=1}^{j-1}\exp\left(-\frac{t_{o}^{j}-t_{o}^{k}}{\tau_{m}}\right), \end{array}$$

and $\partial V\left(t_{o}^{j}\right)/\partial w_{i}$ can be solved by Equation (7), and $\partial t_{o}^{j}/\partial V\left(t_{o}^{k}\right)$ with $t_{o}^{k}<t_{o}^{j}$ can be solved by Equation (8).

Note that each actual output spike time $t_{o}^{j}$ before the *t*_*err*_ is within the tolerable window of the corresponding desired spike time $t_{d}^{j}$, and there is usually a slight deviation between $t_{o}^{j}$ and $t_{d}^{j}$. So the weight modification strategy based on Equation (6) may exacerbate this deviation after multiple updates, resulting in more unnecessary adjustments. In order to address this, in the actual weight adjustment, we substitute $t_{o}^{j}$ for $t_{d}^{j}$ in Equation (6) through Equation (11) and give a scaling factor *S*_*r*_ (> 0) to the second term of Equation (6) to control the weight updating at $t_{d}^{j}$ (< *t*_*err*_) not excessively (the detailed analysis is presented in 4), which is proven to be meaningful and vital by experiments.

#### 2.2.2. Weight Decrement at Undesired Output Spike Times

When there is a spike fired outside the tolerable window (error type a) or there is more than one spike fired inside the same tolerable window (error type b), the contributory synaptic weights should be weakened to prevent the extra spike. Instead of utilizing all the past firing spikes (actual or desired) like the case of weight increment, for error types a and b, synaptic weight decrement depends only on the error time *t*_*err*_, i.e., the scaling rate *S*_*r*_ is set to zero. As a result, the second term in Equation (6) is removed, and the updating rule at undesired output spikes is defined as

(12) $$\begin{array}{l} \Delta w_{i}=-\lambda_{2}\frac{dE}{dw_{i}}=-\lambda_{2}\frac{dV\;\left(t_{err}\right)}{dw_{i}}\approx -\lambda_{2}\frac{\partial V\;\left(t_{err}\right)}{\partial w_{i}}, \end{array}$$

where λ_2_ > 0 is the learning rate which defines the size of the weight decrement. ∂*V*(*t*_*err*_)/∂*w*_*i*_ is solved by Equation (7).

**Figure 2:**
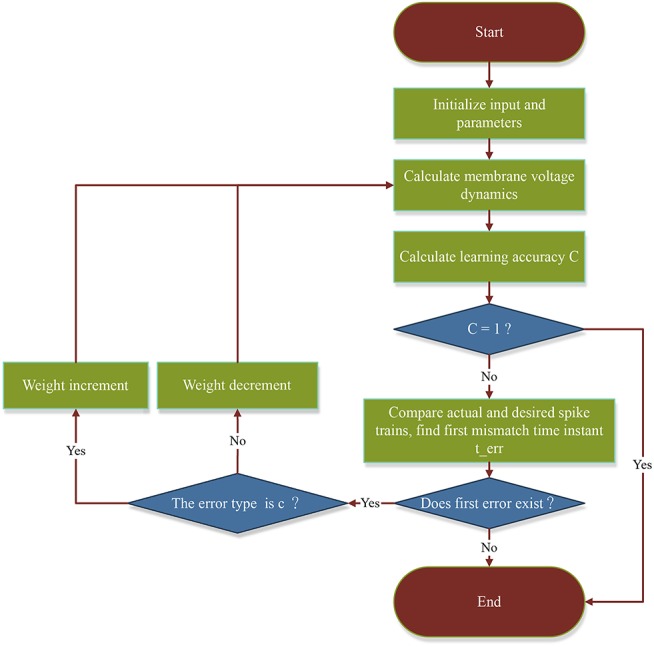
The flowchart of the algorithm FE-Learn.

The intention of removing the second term in Equation (6) is to avoid disturbing the properly emitted output spikes before *t*_*err*_. How this affects the previously emitted spikes is explained in 4. To better illustrate the process of the proposed FE-Learn algorithm, we give a flowchart in Figure 2.

### 2.3. Metric of Learning Performance

The correlation-based metric *C* defined in Schreiber et al. (2003) is adopted in the next experiments to evaluate the learning performance of the learning algorithm, and it was also used in Ponulak and Kasiński (2010) and Taherkhani et al. (2015a). *C*(0 < *C* < 1) represents the similarity degree of two vectors, and the larger the value of *C*, the higher the similarity between the two vectors. The metric is defined in the following equation:

(13) $$C=\frac{\upsilon_{d}\cdot \upsilon_{o}}{\left|\upsilon_{d}||\upsilon_{o}\right|},$$

where ***υ***_***o***_ and ***υ***_***d***_ are vectors which are the convolution (in discrete time) of actual and desired output spike trains by a symmetric Gaussian filter given as *f*(*t*, σ) = exp(−*t*^2^/2σ^2^), respectively. The parameter σ determining the width of the filter is set to 2 in this article. And ***υ***_***d***_ · ***υ***_***o***_ represents the dot product of the two vectors, while |***υ***_***d***_| and |***υ***_***o***_| are the Euclidean norms of them, respectively.

## 3. Simulation results

Next, we conduct extensive experiments to explore the influence of different parameters with different values on the learning performance of the FE-Learn. Moreover, the robustness in the face of noise of different intensities is tested, and finally, FE-Learn is applied to a practical speech recognition task.

### 3.1. Performance Evaluation of FE-Learn

The effects of several important parameters on learning performance are investigated in this section, including the time duration of spike trains, the number of synaptic inputs, and the firing rates of input and output spike trains. We compared the FE-Learn against ReSuMe and SPAN. In these simulations, the time constant of the membrane potential and the synaptic currents, τ_*m*_ and τ_*s*_, are set to 10 and 2.5 ms, respectively. And the firing threshold and the time step are set to 1 mV and 1 ms, respectively. The synaptic weights are randomly initialized by the Gaussian distribution *N*(0.01, 0.01). Twenty trials with different input and desired output pairs are conducted for each experiment.

#### 3.1.1. Effect of the Time Duration

In this section, the learning neuron has 400 synaptic afferents. The aim is to train the neuron to reproduce a desired spike train with a time duration of 200 ~ 3,000 ms and the length of the interval is 200 ms. Before each training trial, the desired output is a spike train with a firing rate of 100 Hz, and input spike trains with a firing rate of 10 Hz are generated according to the homogeneous Poisson processes. During each training, the maximum value of *C* and the running time required to reach it are recorded. After 20 training trials, the average values of all maximum *C* and corresponding running times are reported.

**Figure 3:**
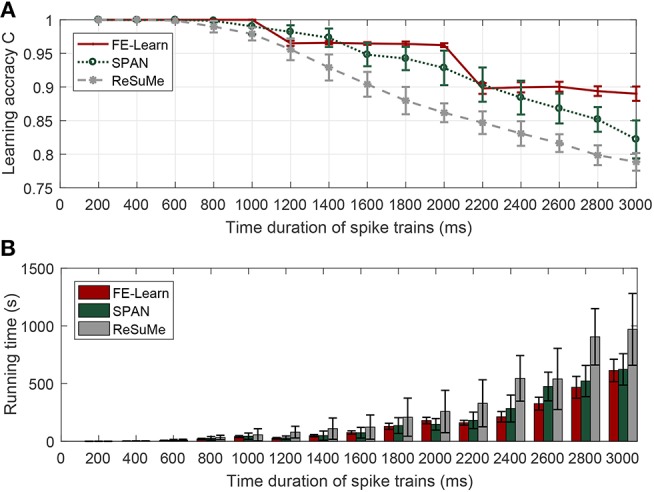
Effect of the time duration of spike trains on learning performance. When the time duration of spike trains is in [200, 1,000], [1,200, 2,000], [2,200, 3,000] ms, the corresponding width of the tolerable window is 1, 3, and 5 ms, respectively. Learning accuracy comparison of FE-Learn, SPAN, and ReSuMe under different time duration of spike trains **(A)**, running time comparison of FE-Learn, SPAN, and ReSuMe under different time duration of spike trains **(B)**.

Figure 3A shows the variation trend in learning accuracies of FE-Learn, SPAN, and ReSuMe. The learning accuracies of the three algorithms can reach one when the time duration of spike trains varies from 200 to 600 ms, but when the time duration exceeds 800 ms, the learning accuracies of SPAN and ReSuMe start to decline, and the learning times increase gradually. Meanwhile, the learning accuracy of FE-Learn is limited by the width of the tolerable window ε, so it can keep constant at 1 when ε = 1, *C* ≈ 0.96 when ε = 3 and *C* ≈ 0.89 when ε = 5, and the learning accuracy drops significantly when the width of the tolerable window changes. Under the same width of the tolerable window, the learning time increases with the increase of spike train length. The general trend is that FE-Learn can obtain higher learning accuracy than SPAN and ReSuMe with less time.

#### 3.1.2. Effect of the Number of the Synaptic Inputs

The effect of the number of the synaptic inputs is investigated in this section, and it varies from 100 to 500 with an interval of 50. The time duration of the spike trains is set to 800 ms. The desired output spike train with a firing rate of 100 Hz and input spike train with a firing rate of 10 Hz are generated according to the homogeneous Poisson processes at the beginning of each training trial.

**Figure 4:**
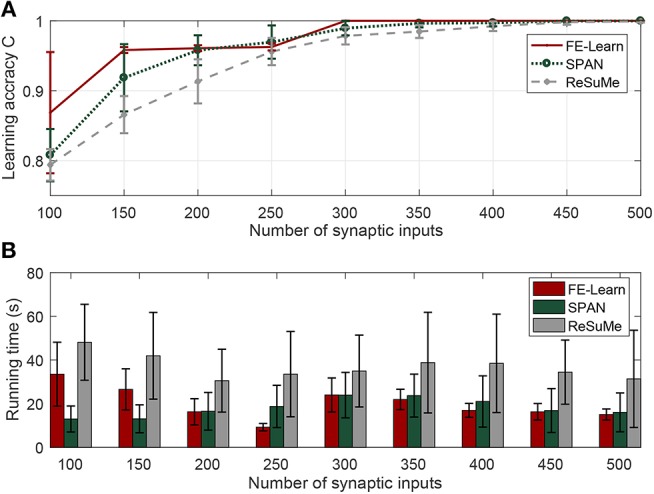
Effect of the number of the synaptic inputs on learning performance. When the synaptic input is in 100, [150, 250], [300, 500], the corresponding width of the tolerable window is 5, 3, and 1, respectively. Learning accuracy comparison of FE-Learn, SPAN, and ReSuMe with different number of the synaptic inputs **(A)**, running time comparison of FE-Learn, SPAN, and ReSuMe with different number of the synaptic inputs **(B)**.

Figure 4 shows the experimental results. As shown in Figure 4A, a small number of synaptic inputs lead to a low learning accuracy for both SPAN and ReSuMe—for instance, the learning accuracy of SPAN is only 0.81 and for ReSuMe it is 0.79—when the neuron is trained with only 100 synaptic inputs, but SPAN takes a very short time, and although FE-Learn with ε = 5 takes more time, it can achieve higher accuracy. When the number of synaptic inputs is greater than or equal to 300, the width of the tolerable window of FE-Learn is set to 1 ms. Then, the learning accuracy of it can reach 1, while the learning accuracies of SPAN and ReSuMe slowly increase to 1 with the increase of the number of synaptic inputs. Additionally, under the same width of the tolerable window, the learning time of FE-Learn can decrease with the increase of the number of the synaptic inputs. In short, FE-Learn performs better than ReSuMe both in terms of accuracy and running time, and obtains higher accuracy than SPAN with comparable time.

#### 3.1.3. Effect of the Firing Rate

**Figure 5:**
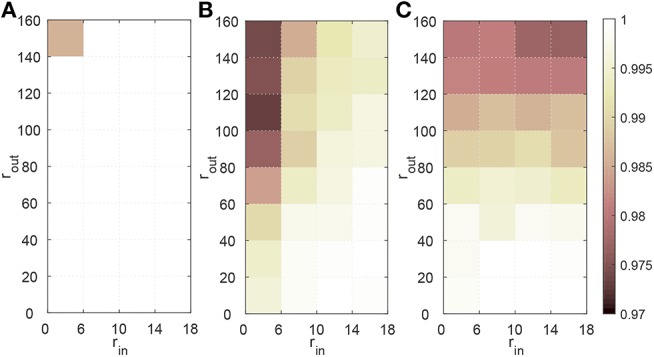
Effect of the firing rate of the spike trains on learning performance of FE-Learn **(A)**, SPAN **(B)**, and ReSuMe **(C)**. All parameters except the firing rates of input spike trains *r*_*in*_ and the desired output spike trains *r*_*out*_ are fixed. The width of the tolerable window ε is set to 1.

The effect of the firing rate of the spike trains is evaluated in the following experiments. For the input spike trains, the firing rates (*r*_*in*_) are varied from 6 to 18 Hz with an interval of 4 Hz, while for the desired output spike trains the firing rates (*r*_*out*_) vary from 20 to 160 Hz with an interval of 20 Hz. The time duration of the spike trains is 800 ms and the amount of the synaptic inputs is set to 400. In each trial, the learning continues until the algorithm converges and the averages of the maximum obtained *C* from 20 trials are reported in Figure 5.

From Figure 5A, the learning accuracy of FE-Learn can achieve 1 except when the firing rates of the input spike train and the desired output spike trains are 6 and 160 Hz, respectively, but even in this worst case, the accuracy still reaches 0.986. However, the performances of SPAN and ReSuMe become worse with the decrease of *r*_*in*_ and the increase of *r*_*out*_, and their lowest accuracies are about 0.97, as shown in Figure 5B.

### 3.2. Robustness to Noise

In this section, the robustness of the neuron trained by FE-Learn and ReSuMe is investigated. The neuron has 400 synaptic inputs. The time duration of the input and expected spike trains is set as 500 ms, both of which are Poisson spike trains, and the firing rates of them are 10 and 100 Hz, respectively. After deterministic training, the response reliability of the neuron is considered in the case of adding background noise on the membrane potential and adding jittering noise on the input pattern.

#### 3.2.1. Robustness to Background Noise on the Membrane Potential

After training, the membrane potential of the trained neuron is affected by background Gaussian white noise with mean 0 and variance σ_*b*_ ∈ [0.03, 0.33] mV in this case. The variance interval is 0.03 mV, and for every value of σ_*b*_, 20 independent experiments are conducted. The metric *C* is still used to measure the similarity of the actual output and desired output.

**Figure 6:**
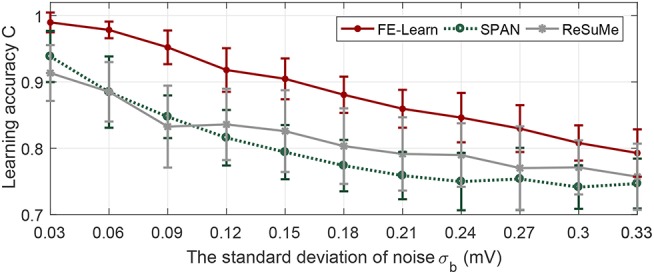
Antinoise capability of FE-Learn, SPAN, and ReSuMe against background voltage noise. The width of the tolerable window ε is set to 1.

As shown in Figure 6, the learning accuracies of the three algorithms decrease with the increase of noise intensity. However, the correlation metric *C* achieved by the neuron trained by FE-Learn is consistently higher than that of SPAN and ReSuMe, confirming that the neuron trained by FE-Learn is more robust when encountering background noise.

#### 3.2.2. Robustness to Jittering Noise on the Input Pattern

In this case, a Gaussian jitter with mean 0 and variance σ_*j*_ ∈ [0.2, 2] ms is added to each input spike after deterministic training. In addition, every spike of the noisy input pattern may be randomly deleted with a probability of 0.05 while some new spikes may be randomly added into the noisy input pattern, which are generated by a 1 Hz homogeneous Poisson process. Just as before, the correlation measure *C* of the distance between the actual and the desired output spike sequences is calculated.

**Figure 7:**
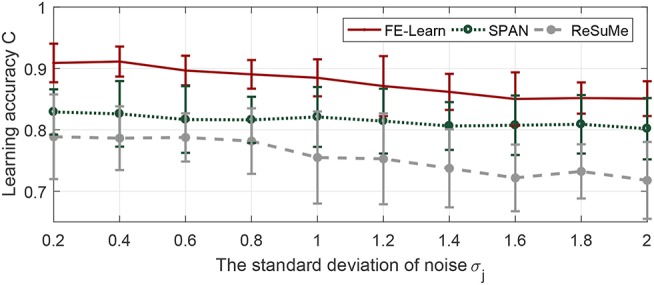
Antinoise capability of FE-Learn, SPAN, and ReSuMe against jittering noise. The width of the tolerable window ε is set to 1.

As can be seen from Figure 7, with the increase of the noise intensity, the correlation between the actual and the desired output spike trains shows a gradual downward trend, but for FE-Learn, it stays about 0.05 and 0.1 higher than that of SPAN and ReSuMe, respectively. Unlike before, SPAN performs better than ReSuMe when exposed to jitter noise. However, neurons trained by the FE-Learn have better anti-noise performance against jitter noise than either of them.

### 3.3. Effect of Learning Parameters

The width of the tolerance window ε and the scaling rate *S*_*r*_ are two important parameters of FE-Learn. We conduct experiments to explore the influence of them on learning efficiency and robustness of FE-Learn. Then we give a spatiotemporal spike pattern recognition experiment, and show the effect of ε on the testing performance.

#### 3.3.1. Effect on Efficiency

**Figure 8:**
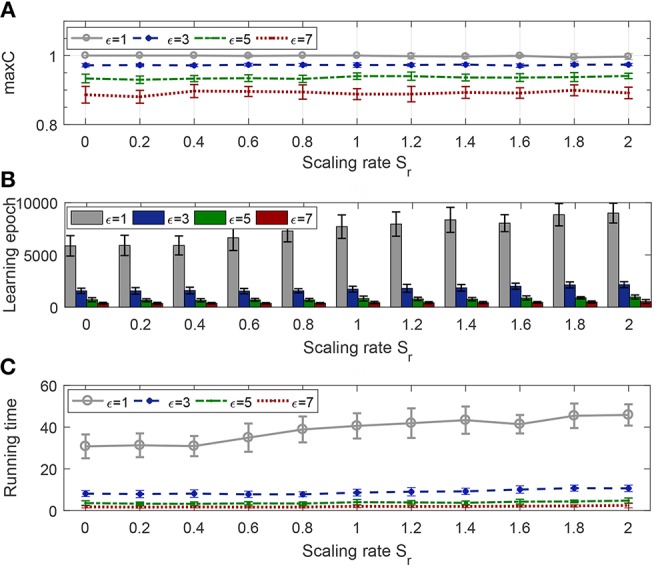
Effect of tolerance window width and scaling rate on learning efficiency. The evaluation index includes learning accuracy **(A)**, the number of epochs **(B)**, and the running time **(C)**.

In this section, the learning neuron has 400 synaptic afferents, and the time duration is 800 ms. Input pattern and target pattern are generated as in the previous experiments with a firing rate of 10 and 400, respectively. The scaling rate varies from 0 to 2 with an interval of 0.2, and the width of the tolerance window has four different values, 1, 3, 5, and 7 (under the condition that time step equals one, width equal to 2 is actually the same as width equal to 1, so there is no need to explore the situation of 2, 4, and 6). For each pair of ε and *S*_*r*_, the learning continues until the algorithm converges and the average of the maximum obtained *C* from 20 trials are reported in Figure 8.

Tolerance window width determines the learning accuracy of convergence, and Figure 8A shows this obviously, and it also shows that no matter what the scaling rate is, the algorithm will eventually converge to the accuracy limited by the corresponding window width. From Figures 8B,C, we can see that, only when the tolerance window width is 1, the time of convergence increases as the scaling rate increases, and is always much higher than other cases, i.e., when the width is greater than 1, the scaling rate has little impact on the convergence speed, and the convergence time is always very small.

#### 3.3.2. Effect on Robustness

The experiment settings are the same as last section, except that the time duration is changed to 500 ms. We add background noise and jittering noise to the network after each training trial.

**Figure 9:**
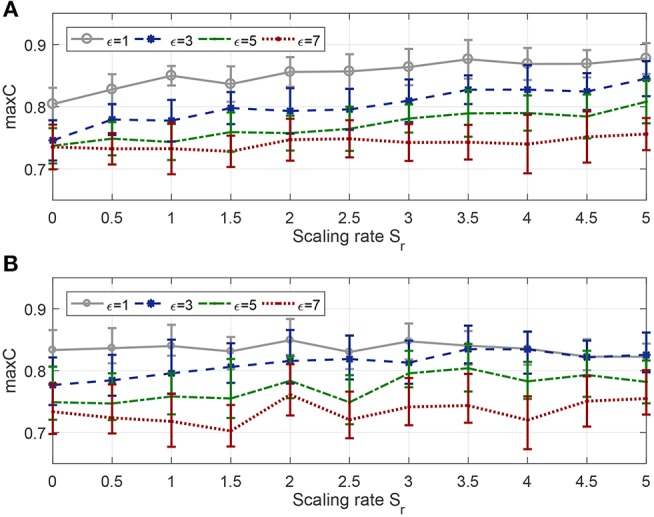
Effect of tolerance window width and scaling rate on robustness. **(A)** Antinoise capability against background noise with standard deviation σ_*b*_ = 0.2. **(B)** Antinoise capability against jittering noise with standard deviation σ_*j*_ = 1.

As seen in Figure 9, whether for background noise or jittering noise, the smaller the tolerance window width, the stronger the noise resistance. From Figure 9A, the antinoise capability against background noise becomes stronger with the increase of scaling rate, but from Figure 9B, the antinoise capability against jittering noise does not change obviously with the change of scaling rate.

Combined with Figures 8, 9, when the window width is greater than 1, FE-Learn can converge rapidly and the convergence speed is not sensitive to the scaling rate, but increasing it can improve the antinoise performance to background noise. When the width is 1, the convergence speed of the algorithm is very slow, and the smaller the scaling rate is, the faster the convergence speed is, but the worse the antinoise performance to background noise is.

#### 3.3.3. Effect of the Width of Tolerance Window on Overfitting

**Figure 10:**
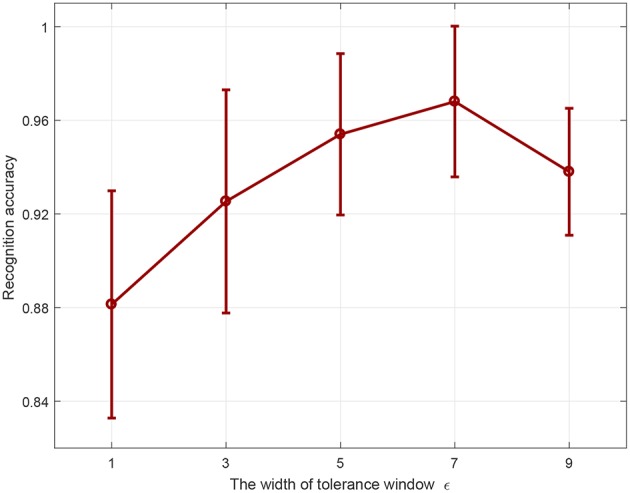
Effect of tolerance window width on overfitting.

In this section, we conduct experiments to investigate the effect of the width of tolerance window on overfitting. Three different spatiotemporal spike patterns are randomly generated with 400 synaptic afferents, all of which are triggered at 5 Hz. The time duration of each spatiotemporal spike pattern is 200 ms. For each spike pattern, 25 samples are generated for training by adding a jitter noise drawn from a Gaussian distribution with a standard deviation of 3 ms, resulting in a training set with 3 × 25 samples. The test set is obtained in the same way. The learning neuron is trained to emit the corresponding desired output spike trains ([5:15:170], [15:15:180], [25:15:190]) in response to the three kinds of spike patterns. When the actual output spike train is most similar to the desired output spike train of a category, then the input pattern is classified into that category. For each ε, the average recognition accuracy on the test set from 20 trials is reported in Figure 10.

As shown in Figure 10, when ε is less than or equal to 7 ms, the classification accuracy on the test set increases with the increase of window width. This is because a smaller window means more rigorous learning on the training set, which will lead to overfitting and reduce the generalization on the test set. For example, when the window width is 7 ms, the mean recognition accuracy on the test set is 96%. However, when the window width is 1 ms, the accuracy is only about 88%. On the other hand, an overly large window will make the training insufficient, thus reducing the recognition accuracy. For instance, the recognition accuracy decreases to 93.80% when the window width is 9 ms. In a nutshell, a relatively large ε generalizes better, and the recognition accuracy on the unseen data is higher.

### 3.4. Classification Task

#### 3.4.1. Spatiotemporal Spike Pattern Classification

**Figure 11:**
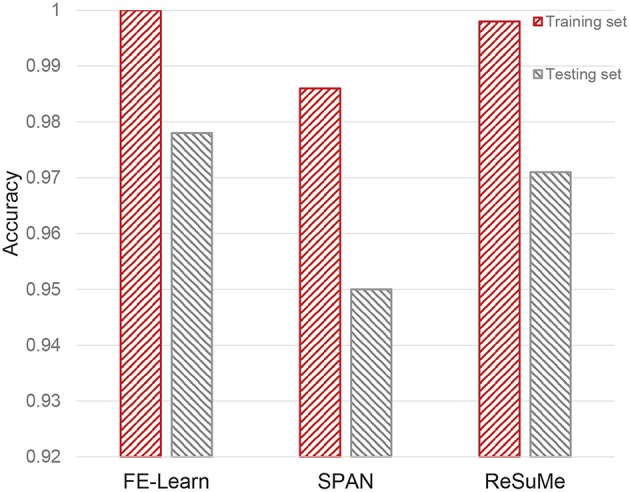
Classification capability of FE-Learn, SPAN, and ReSuMe on spatiotemporal spike patterns.

In this experiment, we investigate the ability of the proposed FE-Learn in classifying spatiotemporal patterns. The setup for the experiment is the same as in section 3.3.3. The aim of the task is to classify three different spatiotemporal spike patterns. Both the training set and test set contain 3 × 25 samples. For each algorithm, after 300 learning epochs on the training set, the classification performance on the training set and test set is tested. The results are shown in Figure 11.

As can be seen from Figure 11, the classification accuracies of FE-learn, SPAN, and ReSuMe on the training set are 1, 0.986, and 0.998 while those on the test set are 0.978, 0.95, and 0.971, respectively. FE-Learn achieves better performance in both the training set and test set. On the other hand, from the respective differences between the training accuracy and the testing accuracy (0.022 for FE-Learn, 0.036 for SPAN, 0.027 for ReSuMe), FE-Learn has a better generalization ability.

#### 3.4.2. Speech Classification

SNNs have great advantages in handling temporally rich signals since they can transform the spatiotemporal information into desired output spike patterns, which means that SNNs are well-suited for realistic tasks such as motion and speech recognition. In order to verify the capability of FE-Learn, the spiking neurons trained by the algorithm are used to conduct a spoken digit classification task. In this work, we investigate the TIDIGITS corpus (Leonard and Doddington, 1993), one of the most commonly used data sets in benchmarking speech recognition algorithms. The utterances of this data set were collected from speakers who come from 22 different dialectical regions and are digit sequences, containing 11 words: “zero,” “one,” ··· , “nine,” and “oh.”

**Figure 12:**
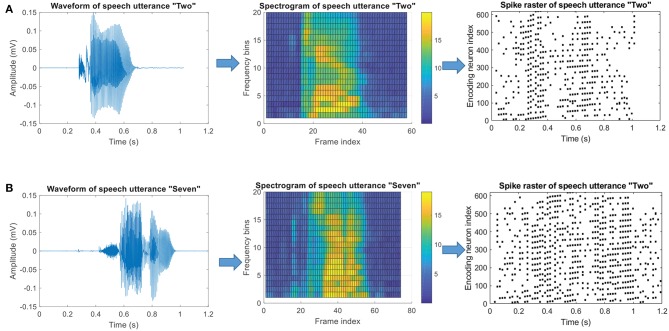
Threshold coding mechanism of speech data. **(A)** The Encoding process of a speech utterance “Two.” **(B)** The Encoding process of a speech utterance “Seven.” The left column shows the original speech waveforms, the middle column shows corresponding spectrograms and the right column shows the final encoded spike pattern.

In this case, the threshold encoding mechanism (Gütig et al., 2009) is adopted to encode the speech data into spike patterns, and the encoding mode is the same as that in Zhang et al. (2019b). Firstly, a Constant-Q Transform (CQT) cochlear filter bank (Pan et al., 2018) is used to filter the original speech waveform to get a spectrogram. Then, the spectrogram is divided into multiple frequency bins. For each bin, a cochlear filter of the corresponding frequency is used to filter it into a series of spikes by recording events that cross thresholds up and down. Finally, the spikes filtered by all cochlear filters are vertically integrated to obtain a complete input spike pattern. Referring to the visualization processing tool of auditory information provided in Dominguez-Morales et al. (2016), a visual representation of this process is given in Figure 12. In the experiment, the training set and test set include 2,464 and 2,486 speech spike patterns, respectively.

**Figure 13:**
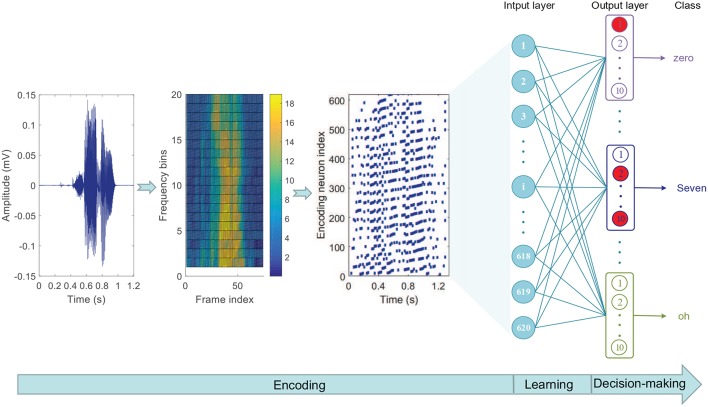
Network architecture of speech classification. The three diagrams on the left show the encoding process of the speech data and the network structure is on the right. The input layer contains 620 neurons and the output layer contains 11 groups of neuron (mullion), corresponding to 11 output categories, and each group is composed of ten neurons. Among the ten output neurons with the same serial number in these 11 groups, the one that emits the most spikes is the “activated” neuron (red circle). The output category belongs to the group with the largest number of “activated” neurons.

The computational model used here is shown in Figure 13. There are eleven groups of output neuron in the classification layer, and each group contains ten neurons, which correspond to the same category. The goal of this experiment is to train the target group of neurons to emit a desired spike train when receiving the input patterns of the corresponding category, and to remain silent otherwise. However, it is not clear how to determine the target output spike train corresponding to each category as each speech digit category contains many different sub-patterns and the differences between these sub-patterns make a fixed desired output spike train impractical. To resolve this problem, a strategy for dynamically determining the target spike train is proposed as follows.

When entering a training input pattern, we record the membrane voltage traces of target neurons and non-target neurons. The desired spike trains *T*_*d*_ and the first wrong time *t*_*err*_ are defined as follows.

1. For the non-target neurons: *T*_*d*_ = ∅.
  - If no spike is generated, no learning is required.
  - If the actual output spike trains *T*_*o*_ ≠ ∅, then the first wrong spike time *t*_*err*_ is the first actual output spike time.
2. For the non-target neurons: *T*_*d*_ is dynamically determined, and suppose *t*_*max*_ is the time instant when the maximum membrane voltage *V*_*max*_ under the threshold is reached. ϑ_*e*_ (< ϑ) is a pre-defined encoding threshold.
  - If no spike is generated, *T*_*d*_ = {*t*_*max*_}, then obviously, *t*_*err*_ = *t*_*max*_.
  - If the actual output spike trains *T*_*o*_ ≠ ∅ and *V*_*max*_ is above the pre-defined encoding threshold ϑ_*e*_, then *T*_*d*_ = *T*_*o*_ ∪ {*t*_*max*_}, *t*_*err*_ = *t*_*max*_.
  - If the actual output spike trains *T*_*o*_ ≠ ∅ and *V*_*max*_ is below the pre-defined encoding threshold ϑ_*e*_, then *T*_*d*_ = *T*_*o*_ and no learning is required.

According to the defined *T*_*d*_ and *t*_*err*_, the corresponding weight updating formula is called for learning. During the test, the output category belongs to the group with the largest number of activated neurons (red neuron shown in the output layer in Figure 13). Moreover, the training strategy with margins in Gütig (2016) is applied in this work. We also test the performance of ReSuMe and SPAN on this task with the same network configuration, encoding method, and training strategy as FE-Learn.

As shown in Table 1, the spiking convolutional neural network (Tavanaei and Maida, 2016) and the deep recurrent network (Neil and Liu, 2016) perform well in this speech recognition task, and they can obtain an accuracy of 96 and 96.1%, respectively. However, compared with their complex network structures, the computational model we used here is very simple while the accuracy of our method is higher than others. As shown in Table 1, the single layer spiking neural network with the proposed FE-Learn algorithm obtains an accuracy of 96.42%, which is superior to other biologically motivated baselines, as well as ReSuMe and SPAN with the same network structure, encoding scheme, and training strategy. The excellent performance of FE-Learn shows its great potential in practical application.

**Table 1:** Comparison of speech recognition performance among several frameworks.

**Model**	**Accuracy**
Spiking CNN and HMM (Tavanaei and Maida, 2016)	96.00%
Single-layer SNN and SVM (Tavanaei and Maida, 2017)	91.00%
AER Silicon Cochlea and Deep RNN (Neil and Liu, 2016)	96.10%
Liquid State Machine (Zhang et al., 2015)	92.30%
AER Silicon Cochlea and SVM (Abdollahi and Liu, 2011)	95.58%
Auditory Spectrogram and SVM (Abdollahi and Liu, 2011)	78.73%
Single-layer SNN with SPAN	91.22%
Single-layer SNN with ReSuMe	93.52%
Single-layer SNN with FE-Learn	96.42%

**Figure 14:**
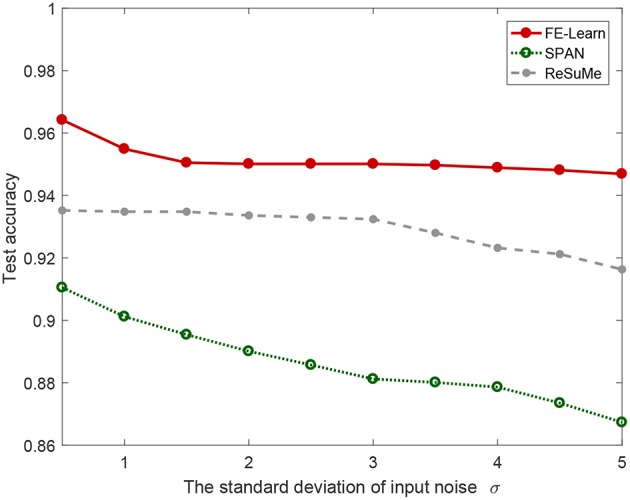
Speech recognition performance of FE-learn, SPAN, and ReSuMe in the test set in the face of input noise.

Additionally, in order to investigate the performance of FE-Learn in more complex cases, we also conduct speech classification experiments of the three algorithms with different input noise intensities. The standard deviation of jitter noise added to the input spike pattern increases from 0.5 to 5 ms with an interval of 0.5 ms. As shown in Figure 14, the classification accuracy of the proposed FE-Learn is 94.69% even when the noise intensity is 5 ms, which is much higher than ReSuMe and SPAN with the same noise level. Therefore, the robustness of the FE-Learn is better than ReSuMe and SPAN in practical application.

## 4. Discussion

In this section, we first analyze the difference between the three algorithms and explain the role of the parameter *S*_*r*_ through a concrete example. Then we figure out the reasons that contribute to FE-Learn's better performance over ReSuMe and SPAN in accuracy, computation time, and generalization.

**Figure 15:**
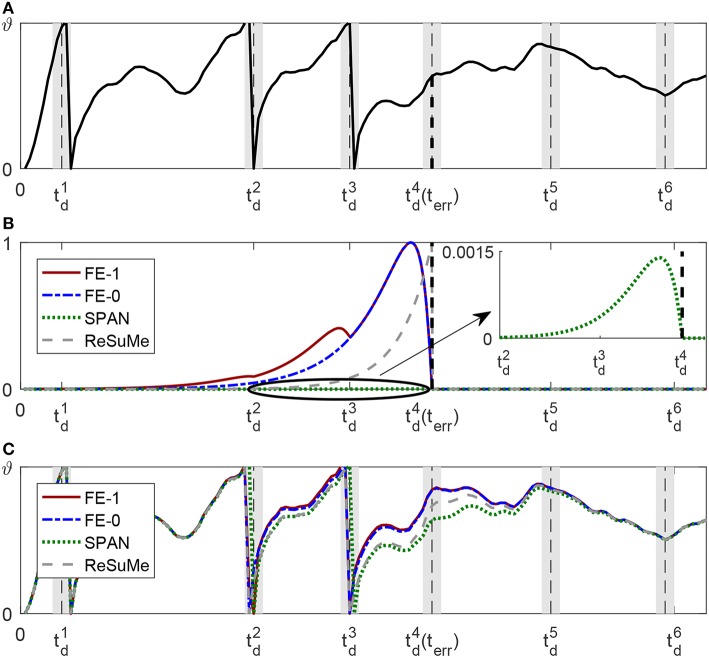
Comparison between FF-learn, SPAN, and ReSuMe for one weight updating. The neuron has been trained to elicit the first three spikes in corresponding tolerable windows, which are represented by the gray shadow region, the desired spike times are located at the middle of the windows. The black vertical dashed lines represent the first wrong time *t*_*err*_. **(A)** Membrane dynamics of the neuron before this learning. **(B)** Synaptic learning curves of FE-Learn with scaling rate *S*_*r*_ = 1 (red solid line) FE-Learn with scaling rate *S*_*r*_ = 0 (blue dotted line), SPAN (green dotted line), and ReSuMe (gray dashed line). **(C)** Membrane dynamics of the neuron after one learning using different rules.

The membrane potential curves before and after a single weight updating have been shown in Figures 15A,C, respectively. In Figure 15B, the synaptic learning curves depict the spike-timing dependence of weight adjustment at time *t*_*err*_. ReSuMe has an exponential learning curve (the gray dashed line), which means that the closer the input spike time is to *t*_*err*_, the larger the synaptic weight update is. However, due to the existence of the time constants of the membrane voltage and synaptic current, the input spike closest to *t*_*err*_ does not make the largest contribution to the membrane voltage at *t*_*err*_, so the learning of ReSuMe does not serve the aim very well. As for SPAN, we depict its spike-timing dependence curve (green dotted line) of weight adjustment with α-kernel in Mohemmed et al. (2012) at time *t*_*err*_. From Figure 15A, each actual output spike time before the *t*_*err*_ is within the tolerable window of the corresponding desired spike time. Accordingly, the convolution of the error is very small, resulting in very little weight change at *t*_*err*_. The shape of the learning curve is determined by the convolution kernel, and the inconsistency between the convolution kernel and the current kernel of the neuron model can also lead to mismatching between the weight change of the synaptic and its potential contribution.

As we already know, FE-learn with *S*_*r*_ = 1 utilizes all the spike times before *t*_*err*_ to calculate weight increment, so the learning curve of it has multiple crests compared with that of FE-learn with *S*_*r*_ = 0 which has one crest. It means that the former would promote the synaptic weights whose input spikes happened before $t_{d}^{3}$ with a larger amount, but for those spikes fired between $t_{d}^{3}$ and $t_{d}^{4}$, the weight updates are the same (the red solid line and the blue dashed line coincide). As shown in Figure 15C, the membrane potential at $t_{d}^{4}$ is successfully raised in all cases, and the spike times before $t_{d}^{4}$ are pushed forward a little bit. But for FE-learn with *S*_*r*_ = 1, this is more obvious than others because of the greater weight updates and thus the greater voltages at these times, which means that it is more robust to noise disturbance. However, an overly strong weight update may cause the previous output spikes to be removed from the corresponding tolerable windows, so the appropriate strength of weight adjustment at previous desired spike times which is controlled by the scaling factor *S*_*r*_ is crucial. As for the case of weight decrement, we only want to reduce the membrane voltage at *t*_*err*_, but do not want the previously correctly emitted spikes to be affected, so setting *S*_*r*_ to zero is reasonable.

As shown in the experimental results, FE-Learn achieves a higher learning accuracy with less training time and has a better generalization. First of all, the reason for the high accuracy of our method is that our method follows the BPBA (Bigger PSP, Bigger Adjustment) (Xu et al., 2013a) principle to effectively overcome learning interference among multiple desired spikes, while the weight update rules in ReSuMe and SPAN cannot be combined with the BPBA principle. Besides, to improve the efficiency of the program, we have calculated and stored the PSPs (Postsynaptic potentials) of every time step before training. For example, when the time duration is *T*, the time step is *dt* and the number of the synaptic inputs is *N*, storing the calculated PSPs requires *N* · *T*/*dt* storage units. For the three algorithms, the calculation of the neuron dynamics and weight adjustments are all based on the stored PSPs, and the additional memory costs required by them are very small, implying that they have a similar memory overhead. On the other hand, in each training epoch, ReSuMe makes multiple weight adjustments at each desired and actual firing time, while SPAN changes weight at each time step. However, FE-Learn only makes a weight adjustment once at *t*_*err*_ in one epoch, and the membrane potential after *t*_*err*_ does not need to be calculated in our experiments. This is the reason that FE-Learn requires less computation time. Finally, as the constraint on the tolerable window for spiking loosens, the generalization ability of proposed FE-learn learning is much better than others. This is the reason for the better results in Figure 11.

## 5. Conclusion

The proposed FE-Learn is designed for identifying spatiotemporal spike patterns, i.e., the neuron is trained to output the specific spike sequence for the given input spike pattern. FE-Learn adjusts the synaptic weights at the first wrong output spike time, and only when the trained neuron correctly fires the first spike at the desired time does FE-Learn begin to focus on adjusting the weights to fire the second desired spike. The adjustment of the synaptic weight is proportional to the derivative of the membrane voltage of the first wrong time with respect to the synapse. These three error types described above actually belong to two types: one is at the desired spike time, the other is at the actual spike time. They correspond to the two opposite cases of increasing and decreasing synaptic weights. For the first case, the desired spike times before the wrong spike time are also used to calculate the derivative, but for the second case, only the wrong spike time is used.

Although the proposed FE-Learn has reliable performance in the experiments, the inherent properties of this algorithm make it converge to the narrow window of the desired spike times, and it is difficult to emit a precisely timed spike. Hence we will explore how to balance the width of the window (accuracy) and the learning speed in the next work. Furthermore, extending FE-Learn to multi-layer deep spiking neural networks is another interesting future direction to explore.

## Data Availability

The datasets analyzed for this study can be found in the TIDIGITS speech corpus https://catalog.ldc.upenn.edu/LDC93S10.

## Author Contributions

XL performed the experiments and writing. XL, HQ, YC, and YZ contributed to the experiment's design and interpretation of the results.

### Conflict of Interest Statement

The authors declare that the research was conducted in the absence of any commercial or financial relationships that could be construed as a potential conflict of interest.
